# Supplementary figures and images for: Determination of metformin bio-distribution by LC-MS/MS in mice treated with a clinically relevant paradigm
Source: PLoS One. 2020 Jun 11;15(6):e0234571. doi: 10.1371/journal.pone.0234571 (PMC7289415; doi:10.1371/journal.pone.0234571)

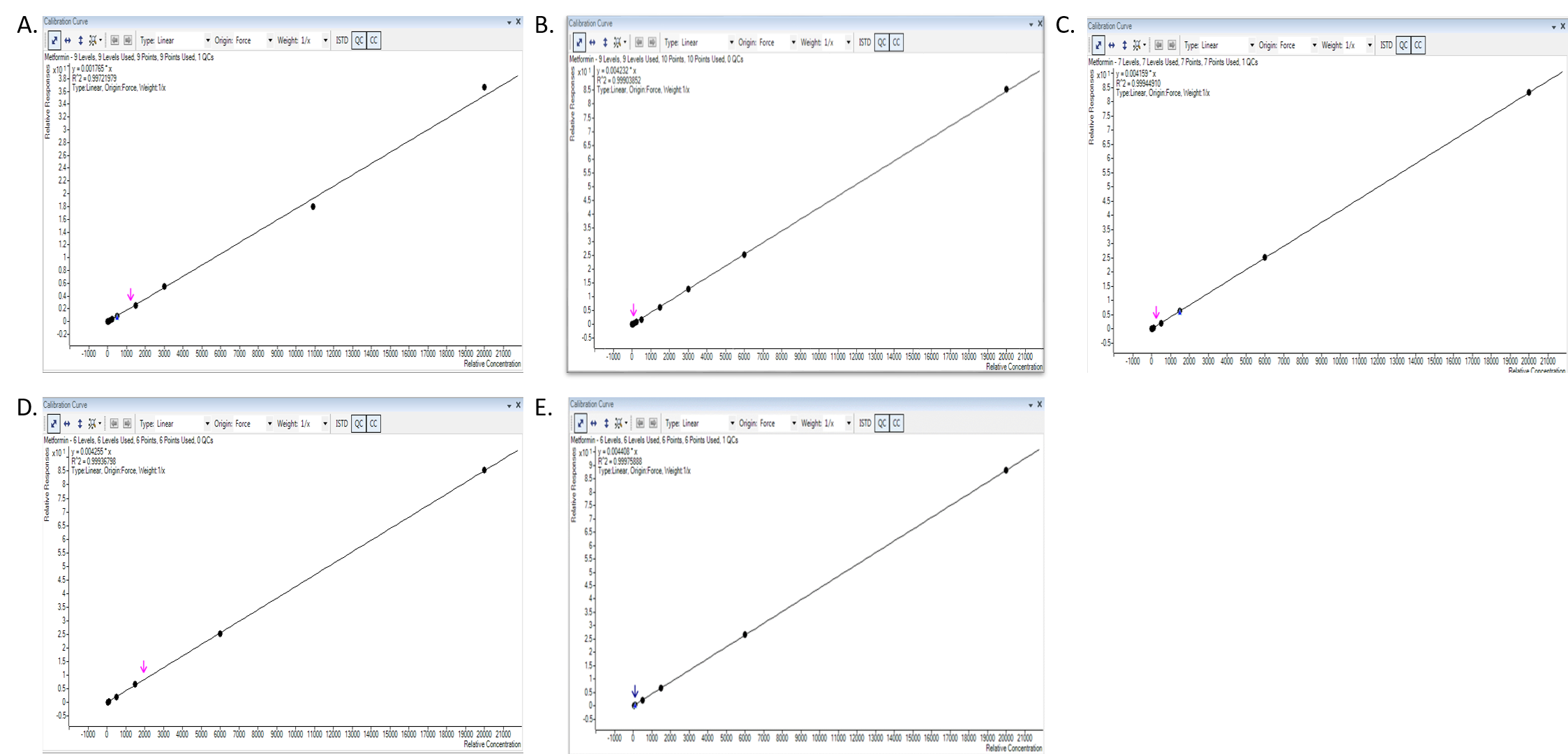

Supplement: S1 Fig — A. Plasma; B. Brain; C. Muscle; D. Liver; E. Kidney. The Y-axis shows the area of the peak (AUC) generated by the MS transition by LC-MS/MS. The X-axis shows the concentration analyzed. Data didn’t deviate from linearity (r2> 0.995) over the tested ranges. Data were fit by linear regression analysis. (TIF) [file pone.0234571.s001.tif]
