# Supplementary material for: Determination of metformin bio-distribution by LC-MS/MS in mice treated with a clinically relevant paradigm
Source: PLoS One. 2020 Jun 11;15(6):e0234571. doi: 10.1371/journal.pone.0234571 (PMC7289415; doi:10.1371/journal.pone.0234571)
Supplement: S1 Table — (DOCX) [file pone.0234571.s002.docx]

| **S 1 Table. Metformin and Metformin-D6 MRM Parameters:** Optimal Values for ESI-MS/MS Detection | | | | | |
| --- | --- | --- | --- | --- | --- |
| **Analyte** | **Precursor Ion🡪 Product Ion (m/z)** | **RT**  **(min)** | **Fragmentor**  **( V)** | **CE**  **(eV)** | **Cell Accelerator**  **(V)** |
| Metformin 1 | 130.2 --> 60.2 | 0.89 | 98 | 13 | 7 |
| Metformin 2 | 130.2 --> 71.2 | 0.89 | 98 | 15 | 7 |
| Metformin-D6 | 136.2 --> 60.1 | 0.89 | 98 | 13 | 7 |
| Metformin 1 is quantifier; metformin 2 is qualifier; metformin-D6 is internal standard; RT= retention time;  CE= Collision energy; m/z= mass to charge; z=1. Min= minutes; V= Volts; eV= electron Volts. | | | | | |
