# Supplementary material for: Determination of metformin bio-distribution by LC-MS/MS in mice treated with a clinically relevant paradigm
Source: PLoS One. 2020 Jun 11;15(6):e0234571. doi: 10.1371/journal.pone.0234571 (PMC7289415; doi:10.1371/journal.pone.0234571)
Supplement: S2 Table — (DOCX) [file pone.0234571.s003.docx]

| **S2 Table. Summary of Linear Regression from the calibration curve across plasma and tissues.** | | | |
| --- | --- | --- | --- |
| **Tissue** | **Regression**  **equation** | **correlation**  **coefficient (r^2^)** | **Parameters** |
| Plasma | Y=0.001765*X | 0.9972 | WEIGHT: 1/X, Origin: force Type: linear |
| Brain | y=0.004232*X | 0.999 | WEIGHT: 1/X, Origin: force Type: linear |
| Muscle | Y=0.004159*X | 0.9994 | WEIGHT: 1/X, Origin: force Type: linear |
| Liver | Y=0.004255*X | 0.9994 | WEIGHT: 1/X, Origin: force Type: linear |
| Kidney | y=0.004408* X | 0.9998 | WEIGHT: 1/X, Origin: force Type: linear |
| r^2^ values were always >0.9972 across different sample sources. | | | |
