# Supplementary material for: Determination of metformin bio-distribution by LC-MS/MS in mice treated with a clinically relevant paradigm
Source: PLoS One. 2020 Jun 11;15(6):e0234571. doi: 10.1371/journal.pone.0234571 (PMC7289415; doi:10.1371/journal.pone.0234571)
Supplement: S3 Table — (DOCX) [file pone.0234571.s004.docx]

| **S3 Table. Stability of metformin under different storage conditions.** | | | | | |
| --- | --- | --- | --- | --- | --- |
| **Concentration**  **ng/ml** | **Autosampler stability**  **48 hours,**  **room temperature** | **Benchtop stability**  **8 hours** | **Long-term stability**  **82 days at**  **-80 ^o^C** | **Freeze-Thaw Stability** | |
|  |  |  |  | **Cycle 1** | **Cycle 2** |
| 50 | 96.3 ± 11.3 | 92.1 ± 9.6 | 112.5 ± 0.27 | 90.1 ± 7.9 | 89.7 ± 8.6 |
| 3000 | 108.7 ± 7.5 | 95.8 ± 10.2 | 95.6 ± 12.5 | 102.5 ± 6.1 | 99.4 ± 5.9 |
| n=6 at each concentration: Values expressed as % mean accuracy ± SD. | | | | | |
