# Supplementary material for: Determination of metformin bio-distribution by LC-MS/MS in mice treated with a clinically relevant paradigm
Source: PLoS One. 2020 Jun 11;15(6):e0234571. doi: 10.1371/journal.pone.0234571 (PMC7289415; doi:10.1371/journal.pone.0234571)
Supplement: Attachment — Submitted filename: Response to reviewer comments.docx [file pone.0234571.s005.docx]

**Determination of metformin bio-distribution by LC-MS/MS in mice treated with a clinically relevant paradigm**

**Response to editor and reviewer’s comments::**

We greatly appreciate the positive and constructive comments raised by the editor and the reviewers for our manuscript titled: “*Determination of metformin bio-distribution by LC-MS/MS in mice treated with a clinically relevant paradigm*”. We have carefully re-revised our manuscript and responded to the comments posed. Below are our responses and indications of the modifications made in the manuscript in response to the comments. The responses are indicated in the order of appearance in the critic by the reviewer. The *italicized* sections below indicate that the phases/sentences are in the revised version of manuscript. The page and line numbers mentioned in the response document corresponds to the revised manuscript with track changes.

**Editor:**

**Response:** We thank the editor for considering our manuscript and providing suggestions to improve the oveall quality of the article. We have accepted all the following suggestions and made necessary changes as follows:

1. Please ensure that your manuscript meets PLOS ONE's style requirements, including those for file naming. The PLOS ONE style templates can be found at
<https://journals.plos.org/plosone/s/file?id=wjVg/PLOSOne_formatting_sample_main_body.pdf> and
<https://journals.plos.org/plosone/s/file?id=ba62/PLOSOne_formatting_sample_title_authors_affiliations.pdf>

**Response:** We have now followed the instruction provided in the pdf documents above and made necessary changes.

2. At this time, we request that you  please report additional details in your Methods section regarding animal care, as per our editorial guidelines:

(a) Please **state the number of mice used** in the study

**Response:**  For this project we used total 24 C57/B6J mice which included young (3 month) males (n=12) and females (n=12). The information is now added on page 5 line 92 onwards.

(b) Please provide the **name and dosage of the specific anesthetic agent** used during the cardiac puncture

**Response:** We have now added the details in methods section on page 5, line 98 onwards as *–“Isoflurane (#NDC-66794-017-25; Piramal Healthcare, Boston, MA, USA) anesthesia induction was conducted in the open drop chamber. Further, during cardiac puncture anesthesia was maintained using a 15 ml tube nose cone. The procedure of cardiac puncture was carried out without opening the chest using 22-gauge needle attached to 1 ml syringe. The needle was inserted just below left end of sternum, pushing horizontally, towards the heart until blood is noticed in the syringe. The cardiac puncture and blood collection procedure was completed in less than one minute under maintenance anesthesia.”*

(c) Please clarify that the **method of euthanasia** used in the study was cardiac puncture.

**Response:** The methods details are now updated on page 5 line 105 onwards as- *“Immediately after cardiac puncture, the mouse was euthanized with cervical dislocation followed byThen the mice were perfusioned with cold normal saline to remove remaining blood from the organs.”*

**Reviewer #1:**

We thank the reviewer for his/he time reviewing our manuscript and suggesting points to improve this manuscript. We have accepted all the suggestions and added required information in the manuscript as follow-

1. Line No 175: mention the concentrations of QC samples.
**Response:** We have now added the concentrations of LLOQ, 10 ng/ml, LQC, 50 ng/ml, MQC, 1,000 ng/ml, HQC, 3,000 ng/ml in the text as well in Table 1.

2. Line No 190-194: As per the Bioanalytical Method Validation Guidance for Industry, precision and accuracy is recommended to perform at LLOQ, low, mid and high QC levels.

**Response:** Thank you for the advice. Indeed the LLOQ is one of the evaluations for precision and accuracy. The data is now added to the updated **Table 1**.

3. Line No 229-230: The author need to provide data for long term stability to justify the statement.

**Response:** We appreciate the need for this additional data. As a part of our protocol, we evaluated long-term stability. The details are added in the text as well as **Table S3**. **On page 14 line 293** as - *“The long term stability for plasma at -80^o^C was evaluated and stable for 82 days”* and on **page 15 line 298** as -*“the long term stability studies showed the analyte stable for 82 days at -80 ^o^C (95.6% to 112.5%)”.*

**Reviewer #2:**

Comments on manuscript PONE-D-20-08716, Determination of Metformin Bio-Distribution by LC-MS/MS in Mice Treated with a Clinically Relevant Paradigm by Kiran Chaudhari, Jianmei Wang, Yong Xu, Ali Winters, Linshu Wang, Xiaowei Dong, Eric Y. Cheng, Ran Liu, and Shao-Hua Yang.

The authors developed a LC-MS method for the determination of metformin in mouse plasma, kidney, liver, muscle, and brain. The mice were voluntarily treated with metformin in drinking water. The study established steady-state metformin levels in the mentioned tissues and plasma. The topic is interesting and it fits the scope of the journal. However, there are many imperfections and they should be eliminated before a possible publication.

**Response:** We thank the reviewer for taking time to review our manuscript, appreciating the value of our research, and providing valuable suggestions, which have now helped to make it a better presentation of our research. We have addressed all the suggestion provided by the reviewer and incorporated the responses in the manuscript as below,

1) First of all, the declared in abstract “Nonetheless, very few metformin studies using mice have determined metformin concentrations…”. Despite the number of relevant reports is limited, there are published papers on the topic and some of them are focused specifically on metformin determination in mouse plasma and various tissues by LC-MS. Considering that one of the main goals of the manuscript is the demonstration of the new LC-MS method for metformin analysis in biologically relevant samples it is necessary to cite already published papers in introduction and to discuss them later in Results and discussion part. The authors should compare the existing methods with their newly developed procedure and show strong and weak features of the individual approaches. One table summarizing and comparing the main performance parameters of the methods would be very beneficial.

**Response:** We agree with the reviewer that there are studies previously done to assess the metformin levels, mostly in plasma and specific tissues dependent on the disease (primarily cancers) of focus. Thank you for pinpointing the importance of the current method comparing to others. Initially, we followed other papers to do our sample analysis, and the articles focused on PK study in plasma provided useful information in our method development in mouse plasma. However, our focus was to develop a uniform method that can be used to assess metformin in multiple tissue matrices simultaneously. The literature lacks such information. We have now summarized the previously published methods in the **Table 4** as well as added following details on **page 20 line 397 onwards** as follows-

***“Comparison of current method with existing methods***

*Metformin is the most widely prescribed and marketed antidiabetic world-wide. It is not surprising that numerous methods for quantitation of metformin, or combinations existed (Table 4). However, the majority of published methods focused on plasma and few on other tissues such as liver and tumors. There are few papers discussing a robust method applied to different matrix such as brain. Some methods used complicated, error prone sample preparation (e.g. SPE, LLE), while other methods used strong concentrated buffer in LC-MS for good chromatography. However, it was hard to solve low sensitivity and reproducibility issues. In majority of methods studying metformin in tissues, a long run time and other than metformin-D6 as internal standard could have lowered throughput without the ability to avoid matrix effect. Furthermore, none of the papers investigated the mechanism of metformin (biguanide) tautomerism feature, which influences chromatography in LC-MS/MS. The current method is sensitive and high throughput, but simple method to quantitate metformin in various tissues and plasma in a short total run time-3 minutes. Some of the strengths of our methods are as follows. Our method provides full validation covering plasma, kidney, muscle, liver, and brain. A simple and fast protein-precipitation method yielded a highly sensitive and reproducible method for multiple tissues as well as plasma. The use of metformin-D6 as an internal standard is more appropriate compared to previous papers, which fail to use labeled isotope metformin as an internal standard and relied on different chemical compounds with varied chemical properties than that of metformin. Further, we incorporated the ion ratio of both qualifier and quantifier to quantitate metformin in different tissues and plasma. Finally, our methods provide efficiency in the total sample run. The total run-time is only 3 minutes and post-run 1 minute, which minimized overall buffer usage and benefit the LC pump and MS source maintenance.*

(**Table 4 Continued on next page**)

| ***Table 4. Comparison of metformin assessment methods using LC-MS/MS*** | | | | | | | | | | | |
| --- | --- | --- | --- | --- | --- | --- | --- | --- | --- | --- | --- |
| ***Sr*** | ***Sample prep.*** | ***IS*** | ***RT***  ***(min)*** | ***Run***  ***time***  ***(min)*** | ***ion ratio*** | ***Column*** | ***Mobile***  ***Phase A*** | ***Matrix*** | ***Study*** | ***Method***  ***Validation*** | ***Reference*** |
| *1* | *PPE^a^,* | *Metformin-D6* | *0.89* | *3* | *Yes* | *HR Xbridge C18, 50x3 mm,3.5 μm* | *2 mM ammonium acetate* | *Mouse plasma,*  *liver, kidney, muscle, brain,* | *correlation study* | *Yes* | *Current* |
| *2* | *PPE* | *Metformin-D6* | *n/a* | *n/a* | *No* | *Aquasil C18, 2.1x20 mm, 5 μm* | *n/a* | *Mouse plasma, urine, liver, kidney, cage wash* | *Transporter effect to metformin tissue distribution* | *No* | *[1]* |
| *3* | *PPE* | *Metformin-D6* | *n/a* | *14* | *Yes* | *Pursuit PFP,*  *2 x150 mm. 3 μm* | *0.05% FA in water, 100% MPA-0% MPA gradient run Flow rate: N/A* | *Mouse Serum, Liver, tumor* | *dose study* | *No* | *[2]* |
| *4* | *PPE^b^* | *Alogliptin* | *3.68* | *20* | *Yes* | *Monolithic silica RP18, 100 x4.6 mm, 1.15 μm S* | *10mM ammonium formate pH 3.0 80% MPA, 0.4 ml/min isocratic run* | *Human Plasma* | *PK* | *Yes* | *[3]* |
| *5* | *LLE* | *Glipizide* | *1.84* | *6* | *No* | *Peerless Basic C18, 33 × 4.6 mm, 5 μm* | *0.5% FA in water, 20% MPA, 0.6 ml/min isocratic run* | *Human Plasma* | *Bioequivalence study* | *Yes* | *[4]* |
| *6* | *PPE* | *None* | *0.98* | *14* | *No* | *Gemini NX-C_18_ , 100 × 2.1 mm,3 µm* | *10 mM ammonium acetate and 0.1% FA in water, 90%-5% MPA, 0.25 ml/min Gradient run* | *postmortem blood* | *Matrix effect study* | *Yes* | *[5]* |
| *7* | *PPE* | *Diphen-*  *-hydramine* | *2.6* | *3.5* | *No* | *Zorbax SB C8, 150×4.6 mm 5 μm* | *1% FA in water, 30% MPA, 0.5 ml/min, isocratic run* | *Human Plasma* | *PK* | *Yes* | *[6]* |
| *8* | *PPE* | *Moroxydine* | *3.0* | *8* | *No* | *Synergi POLAR-RP, 250 4.6 mm, 4 µm* | *6 mM ammonium acetate and 0.1% FA, 50% MPA, 1.0 ml/min isocratic run* | *Dog plasma* | *PK* | *Yes* | *[7]* |
| *9* | *SPE* | *Metformin-D6* | *3.1* | *4.5* | *No* | *XSelect HSS CN, 150 × 4.6 mm, 5 μm* | *8 mM ammonium formate in water, pH 4.5, 20% MPA, isocratic run* | *Rat Plasma* | *Bioavailability* | *Yes* | *[8]* |
| *10* | *PPE* | *Phenformin* | *1.45* | *13* | *No* | *Zorbax HILIC Plus, 50 × 2.5 mm, 3.5 μm* | *0.1% FA in water, 25% MPA, 0.2 ml/min isocratic run* | *Rat plasma* | *PK* | *Yes* | *[9]* |
| *11* | *n/a* | *n/a* | *n/a* | *n/a* | *No* | *n/a* | *n/a* | *Human plasma and colonic tissue* | *correlation between plasma/colonic tissue* | *No* | *[10]* |
| *12* | *PPE*  *-LLE* | *Propranolol* | *1.0* | *5* | *No* | *Zorbax C18, 50×4.6 mm, 5 μm* | *0.1% FA in water, 60% MPA, 0.5 ml/min isocratic run* | *Human Plasma* | *PK* | *Yes* | *[11]* |
| *13* | *SPE* | *None* | *3.5* | *5* | *No* | *Cyano, 150 × 4.6 mm, 5 μm* | *10 mM ammonium formate, 25% MPA, flow rate: N/A* | *Human Plasma* | *PK* | *Yes* | *[12]* |
| *14* | *PPE* | *Canagliflozin* | *2.65* | *15* | *No* | *Agilent Eclipse Plus C18, 4.6 × 100 mm, 3.5 μm* | *6 mM ammonium formate and 0.1% FA, 0.5 ml/min, 98%-0% MPA, gradient run* | *Rat plasma* | *PK* | *Yes* | *[13]* |
| *15* | *PPE* | *Phenformin* | *n/a* | *n/a* | *No* | *Hypersil BDS C18, 150 mm × 2.1 mm, 5 μm* | *n/a* | *Rat plasma, liver, kidney, intestine, muscle, heart* | *PK & PD* | *No* | *[14]* |
| *16* | *SPE* | *Alogliptin* | *1.45* | *8* | *No* | *Symmetry® C18, 4.6 x 50 mm, 5 µm* | *10 mM ammonium formate and 0.2% FA, flow rate 0.25 ml/min, 5% MPA, isocratic run* | *Human Plasma* | *PK* | *Yes* | *[15]* |
| *17* | PPE | Metformin-D6 | 2.2 | 3.5 | No | Kinetex HILIC, 50 x 4.6 mm, 2.7 μm | 9 mM ammonium formate, FA is water and 80 mM ammonium formate, FA in ACN | Human Plasma and urine | PK | Yes | [16] |
| *18* | *PPE* | *N-despropyl ropinirole* | *7.7* | *15* | *no* | *Xbridge-HILIC BEH, 150x 2.1 mm, 3.5 um* | *15 mM ammonium formate in water, 12% MPA, 0.25 ml/min isoractic run* | *Human Plasma* | *PK* | *Yes* | *[17]* |
| *19* | *PPE* | *Dapagliflozin* | *3.2* | *8* | *no* | *Acquity UPLC HSS Cyano, 100 x 2.1 mm, 1.8 μm* | *0.1% FA in water, 90% -10% MPA, 0.4 ml/min, Gradient run* | *Rat plasma* | *PK* | *Yes* | *[18]* |
| *20* | *PPE* | *None* | *1.2* | *9* | *no* | *Xbridge C18, 2.1x50 mm,3.5μm* | *1 mM ammonium formate with 0.1% FA, 95% -30% MPA, 0.2 ml/min gradient run.* | *Human plasma* | *PK* | *Yes* | *[19]* |
| *^a^ diluted with 2mM ammonium aceate; ^b^ diluted with 10mM ammonium formate; PPE= Protein precipitation Extraction; LLE= Liquid-liquid Extraction; SPE= Solid Phase Extraction; FA= formic acid; PK= Pharmacokinetics study; n/a= Not available; RT= Retention time; MP= Mobile phase; IS= Internal standard.* | | | | | | | | | | | |

2) Page 5, line 100, Sample preparation and P6, L124, Preparation of calibration curve and quality control samples:

Here, it is unclear why the sample preparation procedure differs from the one used for calibration curve. Why amounts of plasma/tissue homogenates are not the same/similar in both cases? Moreover, I expect that metformin-D6 was added in the calibration samples, however, it is not mentioned in the text. Overall, the description of the procedure used for the preparation of calibration curve is incomplete, many other important parameters are missing.

**Response:** Thank you for bringing the missing details to our notice. Now we have edited and added the required information in Preparation of calibration curve and quality control samples:

**page 7- line 140 onwards:** “*All blank tissues or metformin-free tissues (liver, brain, kidney, muscle) for preparation of standard curve and quality control samples were homogenized in 1:15 Milli-Q water using GLH-01 homogenizer and Sonic Ruptor 250 ultrasonic homogenizer (OMNI international the homogenizer company, Kennesaw, GA). All blank plasma or metformin-free plasma were ready for preparation of calibration standards and quality control samples.”*

**Page 7- line 153 onwards***: “To the 40 µl of calibration standards and quality control samples in tissue homogenate or 20 µl of calibration standards and quality control samples in mouse plasma, 50 µl of metformin-D6 (250 ng/ml) was added, and vortexed for 2 minutes, then 300 µl of acetonitrile was added and vortexed for 5 minutes. This mixture was then centrifuged at 16,000 X g for 10 minutes. The supernatant was transferred into 1.9 ml glass autosampler vials (catalog number:03-391-8, 03-391-9, Fisher brand, Fisher Scientific, Pittsburgh, PA) before adding 1.0 ml of mobile phase A (2 mM Ammonium acetate in water). An aliquot of 5 µl of the diluted supernatant was directly injected on LC-MS/MS.”*

To avoid sample volume differences, we used 40 ul-homogenized tissue to achieve data accuracy by minimizing pipetting errors with larger volume sampling. We preferred to use 40 µl plasma; however, we were limited with the available plasma volume. At 20 µl plasma, we were able to repeat experiments if any needed. Further, we observed that 20 µl plasma gave enough signal in instrument within the calibration range.

3) P6, L112, Liquid chromatographic and instrumentation conditions:

(a.) The solvent gradient used (the time profile) is not described clearly.Moreover, 2 mmol/l ammonium acetate in water cannot be considered as a buffer. It is simply a salt of a weak acid and a weak base and buffering capacity of the resulting solution is very low. What is also surprising is the total run time 1.5 minutes, especially considering relatively low flow rate and, at the same time, the size of the column.

**Response:** We thank the reviewer for pointing this out. The mention of total run time as 1.5 minutes was a typing mistake. The 1.5 minute time indicates the MS scan window. The detailed gradient run description is now added. The total run time is 3.0 minutes with a post-run time of 1.0 minute. I agree with the reviewer that more than 2 mM ammonium acetate in water can provide better equivalent results. Here, we used mobile phase A as the dilution solution during sample preparation.

(b.) Unfortunately, the authors did now specified the void time of the column, however, taking into account the size of the column, it is evident that practically no separation of analytes can take place in such short time and metformin is eluted together with other compounds very quickly and close to the void time of the column. Is there any time to re-equilibrate the column before the next injection? One can even ask for what there is a chromatographic column at all.

**Response:** Thank you to mention that. We have now discussed this in the Results and Discussion. The chromatography about void volume is now added on **page 9 line 191 onwards**-

*“We monitored void volume during method development. The extracted samples using protein precipitation method typically contained large amount of organic solvent, which is not compatible with the initial 95% of aqueous mobile phase composition. Hence, we diluted the extract with large volume of mobile phase A to keep highly polar metformin in aqueous solution. To avoid metformin elute in solvent front in void area around 0.7min, we applied high resolution C18 column and 95% 2 mM ammonium acetate solution for 1.0 minute as post-run and initial run to re-equilibrate the column, and applied 95% acetonitrile for 1.0 minute to elute other non-polar interferences or co-eluting compounds if there were any. Re-equilibrium of the column for 1.0 minute in post-run is able to give consistent metformin chromatography at retention time 0.89 min, well separated from void area.”*

**Page 10 line 217 onwards**- *“To further troubleshoot the peak distortion and split observed with both metformin and metformin-d6; we used HPLC-TOF-MS and confirmed that the metformin itself underwent split or tautomerism. The peak distortion has been reported in several papers regardless of the column or mobile phase used (reference in text 22 and 23).. The peak distortion could cause peaks merging into void peak area, and that is also the reason we strived to improve peak shape in method development.”*

(c.) The authors claimed later (in the validation part of the text) that due to the application of very selective MRM regime of MS and rigorous determination and measuremet of two MS transition ratio it was proved that the method was sufficiently selective. This might be the case. However, still, in my opinion it is appropriate to draw attention to the LC method being closer to FIA more than LC approch. In fact, when the authors try to explain the reason for the peak deformation (P7-8, L151-168 and Figure 2) by tautomerization of metformin, the real reason might be in the application of “unusual” chromatographic conditions on the column. Moreover, the authors’ explanation does not sound convinsingly furthermore when they claim the control of the pH by 2 mM ammonium acetate buffer, please, again consider that pH of such solution is not controlled at all. In fact, pH was much better controlled when they used 0.1 % HCOOH in H2O (and the peaks were distorted).

**Response:** When we were developing the method, using FIA without column gave retention time at 0.2-0.3 minute, the solvent front was about 0.7 minute, and metformin retention was at 0.89 minute with the high resolution C18 column which offered a good separation from the void area using only 5% organic solvent as initial mobile phase composition. We have now added more details on **page 10 line 201 onwards** as below- *“During sample preparation, we initially tried 0.1 % HCOOH as dilution solution; we observed random peak split for metformin in study samples. Metformin-D6 also exhibited similar peak split or distortion. Previous reports indicated, majority methods used ammonium formate or ammonium acetate solution, adjusted pH with formic acid as mobile phase (discussed later in Table 4). In pursuit of symmetric peak shape as well as retention time away from solvent front, we decided to use ammonium acetate not only as dilution solution but also as the mobile phase in sample preparation. This adjustment helped push the run further away from the solvent front. Initially, we tried 10 mM ammonium acetate as dilution solution and mobile phase A. Although, the LC system built up salt quickly decreasing the MS signal when injecting multiple samples over time, yet, we always got uniform and sharp peak shapes. Then we narrowed down to enough volume of 2 mM ammonium acetate as dilution solution and mobile phase A obtaining uniform and sharp peaks without compromising instrument performance. Hence, we decided to use 2 mM ammonium acetate as mobile phase and dilution solution. It is worth noting that adjustment of pH with formic acid in ammonium acetate solution could give similar results. In our study, we were able to obtain satisfied signals and good chromatography for the study without a need for pH adjustment.”*

4) P8, L169, Method validation:

Matrix effect is discussed only and no information on recovery is provided. Moreover, the calculation of matrix effect is not described in sufficient detail (there is only a footnote below Table 2 and it is not clear what is meant by “analyte concentration in standard solution”). Thus, the matrix effect and recovery should be explained (procedure used for the calculation) and documented by data in adequate table.

**Response:** We appreciate keen observation by the reviewer and the need to provide the details on recovery. The **Table 2 is now updated** as well as a new description is added on **page 13 line 257** onwards as follows- .

*“The recovery of metformin was determined at three different QC levels (LQC, MQC, and HQC) by comparing the peak area of metformin from extracted QC samples (n=6 for each level) with the peak area of post-extracted blank plasma spiked with metformin at the corresponding concentration. Recovery was measured by following equation,*

*Recovery = (Peak area of metformin in QC sample / Peak area of metformin at same concentration in post-extracted sample) x 100%*

*The effect of matrix constituents over the ionization of analytes and IS was determined by comparing the peak area ratios (metformin/metformin-D6) of extracted plasma QC samples with the peak area ratios (metformin/metformin-D6) of the solution prepared in deionized water at corresponding concentrations. The calculated Relative Standard Deviation percentage (RSD%) variation limit was expected to be less than 15%. This determination was performed at three different concentration levels (LQC, MQC, and HQC, n = 6 each).*

*MF% = (Peak area ratio of analyte/IS in extracted samples at QC concentration levels/peak area ratio of analyte/IS prepared in deionized water at QC concentration levels) x 100%*

*In our study, the matrix effect was evaluated by analyzing three levels of QC samples (LQC, MQC, and HQC) for metformin. The matrix effect for plasma was calculated and lay within the range of 91.2% to 112.9%. The average matrix effect values obtained were < 10% RSD. The extraction recoveries at three concentration levels ranged from 96.6% to 112.8%. The results indicated that the protein precipitation method showed a high recovery for metformin in* ***Table 2****.”*

**And table 2 is updated as continued on next page :**

| ***Table 2. Matrix effect (% MF) and recovery (%) of QC samples (n=6) for metformin in spiked mouse plasma*** | | | | |
| --- | --- | --- | --- | --- |
| *QC level* | *MF(%)* | *RSD%* | *Recovery (%)* | *RSD%* |
| *LQC* | *91.20%* | *2.33%* | *112.80%* | *9.87%* |
| *MQC* | *103.32%* | *2.53%* | *97.90%* | *6.12%* |
| *HQC* | *112.97%* | *6.81%* | *96.60%* | *9.54%* |

RSD%= Percentage relative standard deviation

5) P18, L349, References:

Official abbreviation should be used uniformly for publication names.

**Response:** Thank you for bringing this to our notice. We previously used the endnote PLOSONE style downloaded from endnote website for this manuscript. Now we have switched to the Vancour style of referencing modified as suggested in the author guidelines.

 6. PLOS authors have the option to publish the peer review history of their article ([what does this mean?](https://nam04.safelinks.protection.outlook.com/?url=https%3A%2F%2Fjournals.plos.org%2Fplosone%2Fs%2Feditorial-and-peer-review-process%23loc-peer-review-history&data=02%7C01%7Cshaohua.yang%40unthsc.edu%7Cf8f12ec5b3d240f4684c08d7e68ed56b%7C70de199207c6480fa318a1afcba03983%7C0%7C1%7C637231373453230105&sdata=Gipmnu4F01L8NglQw%2B9CuRiBoOwNitBxdn%2F%2BMIvB%2FFk%3D&reserved=0)). If published, this will include your full peer review and any attached files.

**Response:** Yes, we are open to the idea to publish the peer review history of this article.

While revising your submission, please upload your figure files to the Preflight Analysis and Conversion Engine (PACE) digital diagnostic tool, [https://pacev2.apexcovantage.com/](https://nam04.safelinks.protection.outlook.com/?url=https%3A%2F%2Fpacev2.apexcovantage.com%2F&data=02%7C01%7Cshaohua.yang%40unthsc.edu%7Cf8f12ec5b3d240f4684c08d7e68ed56b%7C70de199207c6480fa318a1afcba03983%7C0%7C1%7C637231373453240105&sdata=Nim9zIpdB0QXxAudmfwk%2FXjqvFM9FotXy%2B70xqsJCUQ%3D&reserved=0). PACE helps ensure that figures meet PLOS requirements. To use PACE, you must first register as a user. Registration is free. Then, login and navigate to the UPLOAD tab, where you will find detailed instructions on how to use the tool. If you encounter any issues or have any questions when using PACE, please email us at [figures@plos.org](mailto:figures@plos.org). Please note that Supporting Information files do not need this step.

**Resposne:** We have now uploaded and converted all the figures using the PACE tool.

**References for Table 4:** These references have been incorporated in the main manuscript in the sequence as they appear.

1.	Higgins JW, Bedwell DW, Zamek-Gliszczynski MJ. Ablation of both organic cation transporter (OCT)1 and OCT2 alters metformin pharmacokinetics but has no effect on tissue drug exposure and pharmacodynamics. Drug Metab Dispos. 2012;40(6):1170-7. Epub 2012/03/13. doi: 10.1124/dmd.112.044875. PubMed PMID: 22407892.

2.	Chandel NS, Avizonis D, Reczek CR, Weinberg SE, Menz S, Neuhaus R, et al. Are Metformin Doses Used in Murine Cancer Models Clinically Relevant? Cell Metab. 2016;23(4):569-70. Epub 2016/04/15. doi: 10.1016/j.cmet.2016.03.010. PubMed PMID: 27076070.

3.	Mohammed Al Bratty HAA, Sadique Akhtar Javed, Keddal G. Lalitha, Mufarreh Asmari, Jessica Wölker & Sami El Deeb Development and Validation of LC–MS/MS Method for Simultaneous Determination of Metformin and Four Gliptins in Human Plasma. Chromatographia. 2017;(80):891-9. doi: DOI 10.1007/s10337-017-3288-0.

4.	Pinaki Sengupta UB, Animesh Ghosh, Amlan Kanti Sarkar, Bappaditya Chatterjee, Anirbandeep Bose & Tapan Kumar Pal LC–MS–MS Development and Validation for Simultaneous Quantitation of Metformin, Glimepiride and Pioglitazone in Human Plasma and Its Application to a Bioequivalence Study. Chromatographia 2009;(69):1243-50. doi: DOI: 10.1365/s10337-009-1056-50009-5893/09/06.

5.	Gergov M, Nenonen T, Ojanpera I, Ketola RA. Compensation of matrix effects in a standard addition method for metformin in postmortem blood using liquid chromatography-electrospray-tandem mass spectrometry. J Anal Toxicol. 2015;39(5):359-64. Epub 2015/03/10. doi: 10.1093/jat/bkv020. PubMed PMID: 25749607.

6.	Chen X, Gu Q, Qiu F, Zhong D. Rapid determination of metformin in human plasma by liquid chromatography-tandem mass spectrometry method. J Chromatogr B Analyt Technol Biomed Life Sci. 2004;802(2):377-81. Epub 2004/03/17. doi: 10.1016/j.jchromb.2003.12.017. PubMed PMID: 15018802.

7.	Zhang X, Peng Y, Wan P, Yin L, Wang G, Sun J. Simultaneous determination and pharmacokinetic study of metformin and pioglitazone in dog plasma by LC-MS-MS. J Chromatogr Sci. 2014;52(1):52-8. Epub 2013/01/08. doi: 10.1093/chromsci/bms204. PubMed PMID: 23293039.

8.	Shah PA, Shah JV, Sanyal M, Shrivastav PS. LC-tandem mass spectrometry method for the simultaneous determination of metformin and sitagliptin in human plasma after ion-pair solid phase extraction. J Pharm Biomed Anal. 2016;131:64-70. Epub 2016/08/16. doi: 10.1016/j.jpba.2016.08.011. PubMed PMID: 27526402.

9.	Govender K, Adamson JH, Owira P. The development and validation of a LC-MS/MS method for the quantitation of metformin, rifampicin and isoniazid in rat plasma using HILIC chromatography. J Chromatogr B Analyt Technol Biomed Life Sci. 2018;1095:127-37. Epub 2018/08/05. doi: 10.1016/j.jchromb.2018.07.041. PubMed PMID: 30077092.

10.	Paleari L, Burhenne J, Weiss J, Foersch S, Roth W, Parodi A, et al. High Accumulation of Metformin in Colonic Tissue of Subjects With Diabetes or the Metabolic Syndrome. Gastroenterology. 2018;154(5):1543-5. Epub 2018/03/13. doi: 10.1053/j.gastro.2017.12.040. PubMed PMID: 29526732; PubMed Central PMCID: PMCPMC6002843.

11.	Mohamed D, Elshahed MS, Nasr T, Aboutaleb N, Zakaria O. Novel LC-MS/MS method for analysis of metformin and canagliflozin in human plasma: application to a pharmacokinetic study. BMC Chem. 2019;13(1):82. Epub 2019/08/07. doi: 10.1186/s13065-019-0597-4. PubMed PMID: 31384829; PubMed Central PMCID: PMCPMC6661773.

12.	Shah PA, Shrivastav PS, Sharma V, Yadav MS. Challenges in simultaneous extraction and chromatographic separation of metformin and three SGLT-2 inhibitors in human plasma using LC-MS/MS. J Pharm Biomed Anal. 2019;175:112790. Epub 2019/07/31. doi: 10.1016/j.jpba.2019.112790. PubMed PMID: 31362250.

13.	Elgawish MS, Nasser S, Salama I, Abbas AM, Mostafa SM. Liquid chromatography tandem mass spectrometry for the simultaneous determination of metformin and pioglitazone in rat plasma: Application to pharmacokinetic and drug-drug interaction studies. J Chromatogr B Analyt Technol Biomed Life Sci. 2019;1124:47-57. Epub 2019/06/10. doi: 10.1016/j.jchromb.2019.05.036. PubMed PMID: 31177048.

14.	Wu B, Chen M, Gao Y, Hu J, Liu M, Zhang W, et al. In vivo pharmacodynamic and pharmacokinetic effects of metformin mediated by the gut microbiota in rats. Life Sci. 2019;226:185-92. Epub 2019/04/07. doi: 10.1016/j.lfs.2019.04.009. PubMed PMID: 30953641.

15.	Abbas Moussa B, Mahrouse MA, Fawzy MG. A validated LC-MS/MS method for simultaneous determination of linagliptin and metformin in spiked human plasma coupled with solid phase extraction: Application to a pharmacokinetic study in healthy volunteers. J Pharm Biomed Anal. 2019;163:153-61. Epub 2018/10/13. doi: 10.1016/j.jpba.2018.09.052. PubMed PMID: 30312887.

16.	Scherf-Clavel O, Kinzig M, Stoffel MS, Fuhr U, Sorgel F. A HILIC-MS/MS assay for the quantification of metformin and sitagliptin in human plasma and urine: A tool for studying drug transporter perturbation. J Pharm Biomed Anal. 2019;175:112754. Epub 2019/07/25. doi: 10.1016/j.jpba.2019.07.002. PubMed PMID: 31336285.

17.	Antonopoulos N, Machairas G, Migias G, Vonaparti A, Brakoulia V, Pistos C, et al. Hydrophilic Interaction Liquid Chromatography-Electrospray Ionization Mass Spectrometry for Therapeutic Drug Monitoring of Metformin and Rosuvastatin in Human Plasma. Molecules. 2018;23(7). Epub 2018/06/30. doi: 10.3390/molecules23071548. PubMed PMID: 29954074; PubMed Central PMCID: PMCPMC6100608.

18.	Paul D, Allakonda L, Satheeshkumar N. A validated UHPLC-QTOF-MS method for quantification of metformin and teneligliptin in rat plasma: Application to pharmacokinetic interaction study. J Pharm Biomed Anal. 2017;143:1-8. Epub 2017/05/26. doi: 10.1016/j.jpba.2017.05.026. PubMed PMID: 28544884.

19.	Dias BCL, Fachi MM, de Campos ML, Degaut FLD, Peccinini RG, Pontarolo R. A new HPLC-MS/MS method for the simultaneous quantification of SGLT2 inhibitors and metformin in plasma and its application to a pharmacokinetic study in healthy volunteers. Biomed Chromatogr. 2019;33(11):e4663. Epub 2019/07/25. doi: 10.1002/bmc.4663. PubMed PMID: 31339572.
